# Midnolin‐proteasome pathway for protein degradation

**DOI:** 10.1002/mco2.450

**Published:** 2023-12-10

**Authors:** Wei‐Lin Jin

**Affiliations:** ^1^ Institute of Cancer Neuroscience, Medical Frontier Innovation Research Center, The First Hospital of Lanzhou University, The First Clinical Medical College of Lanzhou University Lanzhou China

## Abstract

Overexpression of immediate‐early genes (IEGs) has been linked to was associated with cancer progression and prognosis. In a recent study, Gu et al. reported the midnolin‐proteasome pathway, a novel ubiquitin‐independent proteasomal degradation. 1 The study provided the mechanism of rapid degradation for nuclear proteins with high unsteadiness. Targeting the midnolin‐proteasome pathway might be beneficial for cancer therapy.

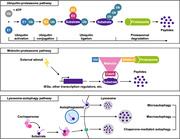

1

Overexpression of immediate‐early genes (IEGs) has been linked to cancer progression and prognosis. In a recent study, Gu et al. reported the midnolin‐proteasome pathway, a novel ubiquitin‐independent proteasomal degradation.^1^ The study provided the mechanism of rapid degradation for nuclear proteins with high unsteadiness. Targeting the midnolin‐proteasome pathway might be beneficial for cancer therapy.

Protein homeostasis, also known as proteostasis, refers to the balance of protein synthesis, folding, and degradation within cells. Disturbances in this process lead to the accumulation of excess, damaged, or unwanted proteins and impaired function of cellular processes and are associated with various diseases, including neurodegenerative diseases and cancer. Understanding the mechanisms of protein homeostasis is essential for uncovering the etiology of specific diseases and developing therapeutic strategies. The ubiquitin‐proteasome pathway and lysosome‐autophagy pathway are two major pathways for protein degradation (Figure 1).^2^, ^3^ Ubiquitin‐proteasome pathway is adenosine triphosphate (ATP)‐dependent and responsible for a majority of protein degradation, especially for short‐lived and regulatory proteins. Ubiquitin required the covalent attachment to the target protein and marked it for degradation by the proteasome. The lysosome‐autophagy pathway, on the other hand, is ATP‐independent and more likely to be involved in long‐lived protein degradation, protein aggregates, damaged organelles, and other cellular components. It involves the formation of autophagosomes, which engulf the targeted cellular components and fuse with lysosomes, where their contents are degraded by lysosomal enzymes. Autophagy in the lysosome‐autophagy pathway could be divided into macroautophagy, microautophagy, and chaperone‐mediated autophagy based on the captured cargo.

**FIGURE 1:**
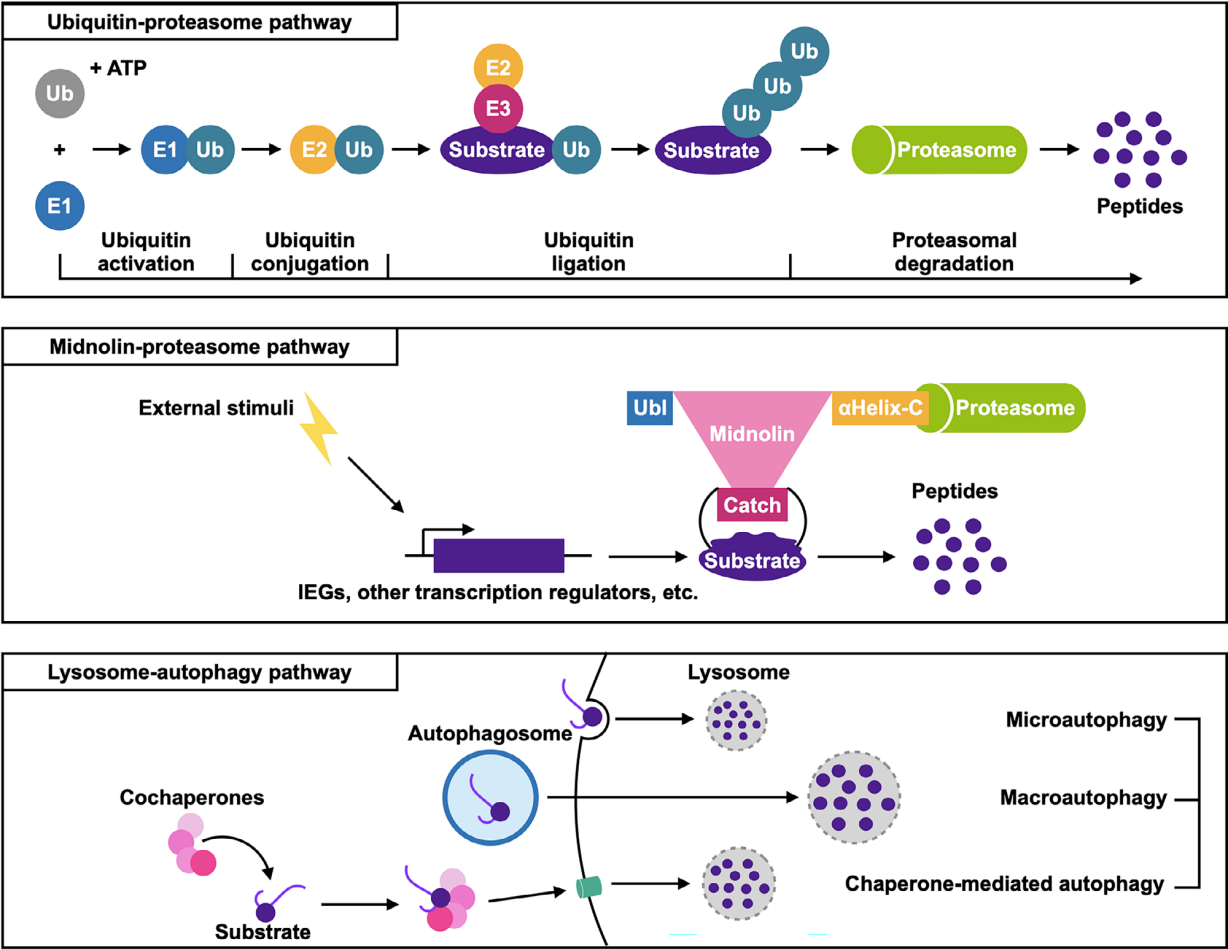
Protein degradation pathways. Degradation of specific proteins can be divided into two categories: proteasome pathway and lysosome‐autophagy pathway. The former could be further classified into the ubiquitin‐proteasome pathway and midnolin‐proteasome pathway, which was a novel ubiquitination‐independent protein degradation pathway discovered by Gu et al.^1^ The latter could be through microautophagy, macroautophagy, and chaperone‐mediated autophagy.

IEGs were defined as a group of genes encoded mainly for transcription factors, cytoplasmic enzymes, and secreted proteins, which had an immediate, rapid, and transient expression (minutes to several hours) upon stimuli.^4^ EGR‐1, c‐FOS, c‐JUN, and c‐MYC were among the most well‐studied and frequently dysregulated IEGs in cancer. They regulated the expression of downstream target genes involved in cell cycle progression, apoptosis resistance, angiogenic factors, and extracellular matrix remodeling, all of which contributed to cancer development and progression. Targeting IEGs was regarded as a vital approach to reducing cancer phenotypes for cancer therapy.^4^

Gu et al. showed that midnolin mediated the ubiquitin‐independent proteasomal degradation of specific proteins encoded by IEGs, such as EGR1, FosB, IRF4, and some other cell type‐specific transcription regulators like NR4A1, c‐FOS, IRF4, PAX8, NeuroD1, and GATA1. In a previous study, Stuxnet, the homolog of midnolin in *Drosophila*, was also demonstrated to promote Polycomb degradation via a ubiquitin‐independent pathway.^5^ It was suggested that midnolin was in the nucleus and nucleolus, contained a ubiquitin‐like domain (Ubl), and was responsible for mRNA transport.^6^ Herein, in Gu et al.’s study, they predicted the structure of midnolin with AlphaFold, indicating that midnolin contained an αHelix‐C, a “Catch” domain, and a Ubl domain. The Ubl domain enabled midnolin‐bound substrates to be degraded by the proteasome, and the “Catch” domain was responsible for “catching” targets for degradation. To verify the capture mechanism, 508 most destabilized proteins uncovered by global protein stability assay with the human open reading frame library (ORFeome) were utilized for AlphaFold prediction and showed a 40% consistency (205/508). Additionally, more unstructured regions might be more prone to be captured by midnolin and formed β strands when binding to the “Catch” domain. Mutations in the hydrophobic residues of the “Catch” domain repressed its ability of midnolin to bind to and degrade its substrates (Figure 1).^1^ The emergence of the midnolin‐proteasome pathway as an alternative protein degradation mode introduced greater diversity and complexity in the regulation of protein turnover and quality control compared to the previously known ubiquitin‐proteasomal protein degradation. Broad‐brush proteomics approaches might be missed in detecting proteins that were extremely short‐lived and increased the risks of bias.

Midnolin (MIDN) was highly expressed in hepatocellular carcinoma (HCC) tumors compared with normal liver.^7^ The high expression of midnolin was associated with poor overall survival and relapse‐free survival of HCC patients. Inhibition of midnolin downregulated Aldh1a1, Ttr, Lpl, Lrp1, Acsl1, Stra6 and upregulated Rbp1 and altered retinoic acid metabolism or lipid metabolism.^7^ ASCL1 was a driver of cancer neuroendocrine phenotype and therapeutic resistance. LRP1 inhibition led to the inhibition of the Notch signaling pathway and tumorigenesis. RBP1 activated oncogenic autophagy and promoted the progression of oral squamous cell carcinoma. MIDN knockdown inhibited liver cancer cell growth and colony formation, which could be rescued by exogenous expression of MIDN. These findings suggested the potential tumor‐promoting effect of midnolin in HCC, but unfortunately, the exact mechanism was not studied. So far, studies of midnolin in other cancer have remained sparse. In cancer, midnolin is needed to adapt to abundant cancer‐related stress, such as oncogene activation, hypoxia, nutrient deprivation, acidosis, high‐demand secretion, metabolite deposition, and endoplasmic reticulum stress. The regulation of midnolin in cancer and roles of midnolin other than mediating protein degradation would guide the investigations on cancer treatment targeting midnolin. Overexpression or knockout/knockdown of MIDN or applying small molecular interventions targeting the midnolin‐proteasome pathway might help modulate an intentional degradation of specific proteins and provide alternative treatment options.

Midnolin might be either the sensor or the effector upon stress, stimuli, energy, or metabolism alteration. Though the exact initiation, targets, and applications of the midnolin‐proteasome system were still being explored, its existence suggested a more diverse landscape of protein degradation, complementing the well‐established ubiquitin‐proteasome pathway. This diversity may offer cells more flexibility in adapting to various physiological and pathological conditions. It is worth noting that the activity of the midnolin‐proteasome pathway might be diverse and region‐specific according to cells and different diseases. Additional studies and investigations are required to validate and expand upon these findings.

## AUTHOR CONTRIBUTIONS

The author has read and approved the final manuscript.

## CONFLICT OF INTEREST STATEMENT

The author declares that he has no competing interests.

## ETHICS APPROVAL STATEMENT

Not applicable.

## Data Availability

Not applicable.
